# Three-Dimensional Objective Evaluation of the Changes in the Alveolar Ridge Before and After Horizontal Bone Augmentation Along with Implant Placement Using Intraoral Digital Scanning: A Prospective Study

**DOI:** 10.3390/jfb16090312

**Published:** 2025-08-28

**Authors:** Naoki Kitamura, Kikue Yamaguchi, Kaiya Himi, Kota Ishii, Motohiro Munakata

**Affiliations:** 1 Department of Implant Dentistry, Showa Medical University Graduate School of Dentistry, 2-1-1, Kita-senzoku, Ota-ku, Tokyo 145-8515, Japan; gd22-n008@dent.showa-u.ac.jp (N.K.); munakata@dent.showa-u.ac.jp (M.M.); 2 Department of Implant Dentistry, Showa Medical University School of Dentistry, 2-1-1, Kita-senzoku, Ota-ku, Tokyo 145-8515, Japan

**Keywords:** dental implant, bone augmentation, guided bone regeneration, volumetric analysis, alveolar ridge, intraoral digital scanning

## Abstract

Implant treatment in the aesthetic regions of the jaw often requires hard and soft tissue augmentation to ensure optimal prosthetic outcomes. Radiological evaluation with cone-beam computed tomography (CBCT) and visual inspection of intraoral photographs are effective for assessing hard tissues but are limited in evaluating soft tissues. This study aimed to objectively evaluate volumetric and dimensional changes of the alveolar ridge, including both hard and soft tissues, following simultaneous horizontal bone augmentation and implant placement using intraoral digital scanning. Intraoral digital scans were obtained at baseline (T0) and at 2 (T1), 6 (T2), and 12 weeks (T3) post-surgery. Scans were superimposed using dedicated imaging software to measure volumetric and cross-sectional changes. Volumetric gain was significant at T1 but decreased significantly from T1 to T2 (*p* = 0.0006) and from T1 to T3 (*p* = 0.0002). Cross-sectional analysis showed significant increases in ridge width at T1 at all measured levels, accompanied by a significant vertical decrease at the alveolar crest from T1 to T2 (*p* = 0.0056) and T3 (*p* = 0.0106).These findings indicate that horizontal augmentation provides initial volumetric gain but is followed by substantial reduction at the crest, suggesting that rigid fixation may enhance stability; however, controlled clinical trials are required.

## 1. Introduction

In recent years, implant treatment has been considered for prosthetic rehabilitation of missing teeth owing to its high long-term survival rates [1]. However, as alveolar bone resorption reportedly occurs after tooth extraction [2,3], some patients undergo alveolar ridge preservation (ARP) or immediate implant placement after tooth extraction [4,5]. In particular, the number of patients requiring hard and soft tissue augmentation has been increasing among patients requiring implant placement in aesthetic areas of the jaw [6,7].

For achieving optimal implant placement considering the prosthetic morphology, implant treatment has recently started to employ technologies that offer three-dimensional (3D) analyses using simulation software. Among these, digital dentistry technologies, such as those classified into static and dynamic guided surgery, have enabled highly precise surgery, including regulation of the implant placement direction [8,9,10,11,12]. However, when considering prosthetic-driven treatment, we often encounter patients who require hard and soft tissue augmentation, such as those with insufficient bone width for implant placement. Nonetheless, the surgical procedure, as well as the extent of hard and soft tissue augmentation, are based on the skill and experience of the operator due to the challenges associated with 3D analysis. Moreover, only limited objective evaluation methods are currently available, such as radiological evaluation with cone-beam computed tomography (CBCT) for hard tissues and subjective visual evaluation by the operator or patient for hard and soft tissues [13,14,15,16,17,18,19,20,21,22,23].

In a radiological evaluation of patients who underwent implant placement with contour augmentation, Buser et al. measured the implant labial bone thickness from CBCT images taken at the 5-to-9-year follow-up [18]. Moreover, Chappuis et al. radiologically evaluated the changes in the alveolar bone in the aesthetic area after tooth extraction by superimposing the data in the Digital Imaging and Communications in Medicine (DICOM) format obtained from CBCT images [22]. However, although these radiological methods are superior in evaluating hard tissues, they have limitations in evaluating soft tissues, including inaccurate depiction of soft tissues and susceptibility to metal artifacts, as well as concerns regarding radiation exposure with time-course CBCT imaging.

In contrast, advances in intraoral scanners (IOSs) have contributed to improved dissemination, accuracy, and trueness over time [24,25,26,27]. Ender et al. evaluated the trueness and accuracy of in vitro digital impressions using multiple IOSs [26,27]. The digital impression of the entire jaw range was found to have a trueness of 32.4 µm to 89.8 µm and an accuracy of 30.1 µm to 58.6 µm. In particular, the digital impression of the partial range of the molar area was reported to have the best trueness value, of 21.9 µm. Moreover, the conventional impression method using polyvinyl siloxane impression material was reported to have a trueness of 9.7 µm, thus indicating that IOSs are clinically well acceptable. Additionally, 3D superimposition methods using special 3D difference analysis software have been reported to be useful for the time-course intraoral monitoring of patients. Additionally, Abad-Corone et al. reported that digital models obtained from IOS are generally processed using CAD/CAM system software, and that the digital mesh resolution at the time of export is important in order to meet clinical requirements [28]. They also reported that high-density meshes enable accurate capture of curved shapes. In recent years, there have been reports on objective evaluation methods for soft tissue changes after soft tissue grafting using digital scanning, and on the assessment of peri-implant tissue changes using intraoral scanning [29,30,31,32,33,34,35]. Badal et al. performed soft tissue grafting in patients with gingival recession and evaluated the changes in the patients’ soft tissues before and after surgery using intraoral digital scanning [36]. They demonstrated that the increased volume and thickness of tissues were measured easily and non-invasively by superimposing the standard tessellation language (STL) data obtained before and after soft tissue grafting.

Gil et al. [37] and Parvini et al. [38] reported a method for three-dimensional assessment of tissue volume changes in the region of interest by superimposing STL data from two time points obtained through longitudinal records of periodontal and peri-implant tissue using digital software. This approach enabled the quantitative evaluation of changes in tissue volume and thickness, which had not been possible with traditional linear measurements using periodontal probes.

However, few studies have objectively evaluated the morphological changes in the alveolar ridge, including hard and soft tissues, before and after bone grafting [39].

Therefore, we performed intraoral digital scanning in patients who underwent horizontal bone augmentation surgery and objectively evaluated morphological changes in the alveolar ridge, including both hard and soft tissues, before and after bone grafting using specialized 3D difference analysis software.

Based on these considerations, we set the following hypotheses:

**H0:**  *There are no significant volumetric or dimensional changes in the alveolar ridge, including hard and soft tissues, before and after horizontal bone augmentation with simultaneous implant placement.*

**H1:**  *Intraoral digital scanning can detect significant volumetric and dimensional changes in the alveolar ridge, including both hard and soft tissues, before and after horizontal bone augmentation with simultaneous implant placement.*

## 2. Materials and Methods

### 2.1. Study Design and Selection Criteria

The participants included patients who underwent implant placement and horizontal bone augmentation for a missing maxillary anterior tooth at the Showa Medical University Dental Hospital Implant Center between March 2023 and July 2024. Participants were given a detailed explanation of the study procedures in writing, and informed consent was obtained. The study protocol was in accordance with the ethical principles based on the Declaration of Helsinki and the Ethical Guidelines for Medical and Biological Research Involving Human Subjects. The Ethics Committee of the Showa Medical University approved this study protocol (approval number 22-243-A; approval date 30 January 2023).

The required sample size was estimated based on the results of a previous study [32], which reported a mean difference of 2.0 with a standard deviation of 2.0.

Assuming a two-tailed test with a significance level (α) of 0.05 and a power (1−β) of 0.80, a minimum of 8 participants per group was determined to be necessary to detect the expected difference with sufficient power.

Participant selection was based on the criteria below.

**Inclusion criteria:**

- Patients who underwent implant placement and horizontal bone augmentation for a missing maxillary anterior tooth at least 2 months after tooth extraction;
- Patients aged ≥20 and ≤65 years at the time of providing consent;
- Patients who themselves provided their written consent to participate in this study.

**Exclusion criteria:**

- Smokers;
- Patients with a history of bone metabolism disorders;
- Patients with a history of receiving antiresorptive drugs;
- Patients with a history of rheumatoid arthritis;
- Patients with a systemic disease, such as diabetes mellitus and heart disease;
- Patients who previously had an implant placed adjacent to the target site;
- Patients with malocclusions, such as tooth crowding or transposition;
- Patients who underwent ARP at the target site;
- Patients who wore removable dentures covering the target site.

### 2.2. Surgical Technique

**Figure 1:**
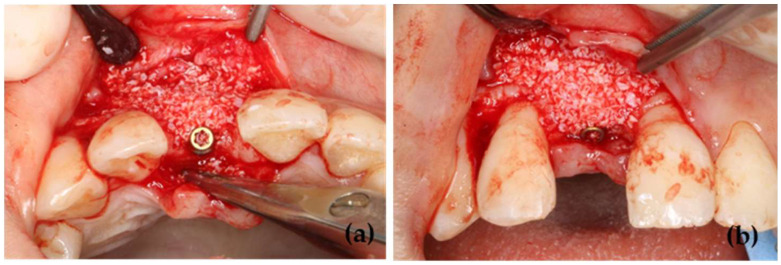
Intraoperative photographs during implant placement and horizontal bone grafting. (**a**) Occlusal view, (**b**) Labial view.

The surgery was performed under intravenous sedation in the Central Operating Room of Showa Medical University Dental Hospital. To prevent postoperative infection, the patients were administered 1000 mg of viccillin intravenously before surgery. After inducing local anesthesia using 2% lidocaine with 1/80,000 adrenaline bitartrate (Ora Inj, GC SHOWA YAKUHIN Co., Ltd., Tokyo, Japan), alveolar crestal and vertical incisions were made, and a gingival mucoperiosteal flap was created. After removing poor granulation tissue and forming an implant cavity according to the usual method, decortication was performed in the labial cortical bone. A bone-level implant body (BLT SLActive Roxoid Loxim, Straumann, Basel, Switzerland) was placed, and bone grafting was performed labially in the bone defect site (Figure 1). A 20:80 mixture of autologous bone and a bovine-derived porous bone substitute (Bio-Oss, Geistlich Pharma AG, Wolhusen, Switzerland) was used in all patients. A porcine-derived type I/III collagen membrane (Bio-Gide, Geistlich Pharma AG, Wolhusen, Switzerland) served as the barrier membrane. The membrane was sutured to the gingival mucoperiosteal flap on the palatal side and extended toward the labial side to cover the grafted bone. Graft stabilization was achieved without the use of fixation pins or tenting screws. The gingival mucoperiosteal flaps on the labial and palatal sides were sutured with 5-0 nylon threads (CROWN JUN, Kono Seisakusho Co., Ltd., Chiba, Japan). The mucoperiosteal flap was closed using a simple interrupted suture technique with the same suture material as described above. Then, after the surgery, the patients were administered 1000 mg of amoxicillin (in four divided doses per day) for 5 days. Additionally, they were instructed to gargle with 0.2% benzethonium chloride solution four times a day for 2 weeks after the surgery. Disinfection and clinical observation were carried out 1 week after the surgery, and the sutures were removed 2 weeks after the surgery. The surgery was performed by implant specialists (M.M and K.Y) with at least 20 years of experience in implant treatment.

### 2.3. Acquisition of Optical Impression and Intraoral Photographs

**Figure 2:**
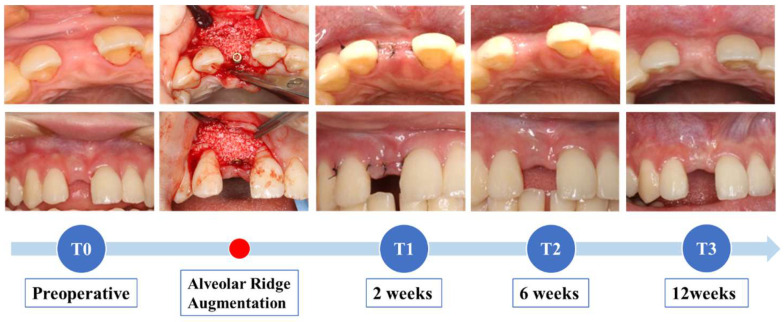
Study protocol.

Intraoral digital scans and intraoral photographs were taken before surgery (T0), 2 weeks after surgery (T1, ±1 week), 6 weeks after surgery (T2, ±1 week), and 12 weeks after surgery (T3, ±2 weeks) (Figure 2). An IOS (Primescan, Dentsply Sirona Inc., Charlotte, NC, USA) was used for intraoral digital scanning. Intraoral digital scanning was performed by the same operator twice for all patients. The range of scanning covered not only the site of the missing tooth but also at least two adjacent teeth. Using the IOS, digital scanning was initiated from the palatal area of the left premolar while maintaining a constant speed in one direction. Handling was performed in the following order: from the palatal area of the anterior tooth to the palatal area of the right premolar, then from the occlusal surface of the right premolar to the occlusal surface of the left premolar, and lastly from the buccal area of the left premolar to the buccal area of the right premolar. Instead of conducting additional baseline scans of typodont or stone models, we refer to published validation studies of the Primescan for trueness and precision metrics. Nulty et al. reported that Primescan achieved an excellent full-arch trueness of 17.3 ± 4.9 µm (closely comparable to high-precision laboratory scanners) and precision within the same range [40]. These figures substantiate the high baseline accuracy of the scanner used, demonstrating that additional calibration scans would likely produce comparable performance.

### 2.4. Digital Measurement of Changes in the Alveolar Ridge in the Region of Interest

**Figure 3:**
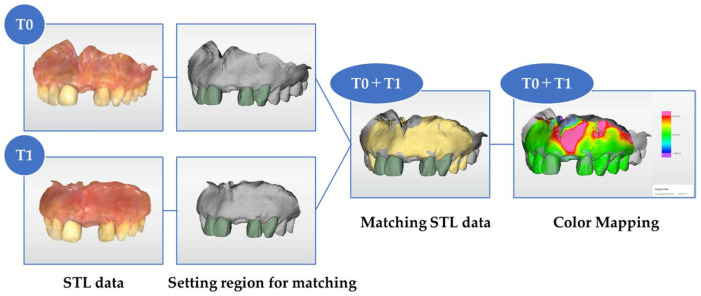
Matching workflow of the standard tessellation language (STL) data before surgery (T0) and after surgery (T1, T2, and T3).

The acquired data were exported in the STL file format after orienting the digital model using dedicated computer-aided design/computer-aided manufacturing (CAD/CAM) software (CEREC SW 5.1.3 Dentsply Sirona Inc., Charlotte, NC, USA). The data obtained after surgery (T1, T2, or T3) were superimposed with the data obtained before surgery (T0) using matching software (Oracheck 5.0.0, Dentsply Sirona Inc., Charlotte, NC, USA) These procedures were performed by a different examiner from the operator who performed intraoral scan. Importantly, the examiner conducting the analysis was blinded to the time points of the scans in order to reduce expectation bias. (Figure 3).

Two adjacent teeth that were not likely to change their surface shape or move over time were selected as the reference regions for superimposition, and the two sets of STL data were superimposed using best-fit alignment.

To determine the temporal changes in the alveolar ridge at T1, T2, and T3 from the baseline (T0), we measured the volumetric changes (mm^3^) in the region of interest, as well as vertical/horizontal dimensional changes in the alveolar ridge (mm). Volumetric and dimensional changes were measured twice by the same experienced tester, and their mean was used in the analysis.

#### 2.4.1. Establishing a Region of Interest

**Figure 4:**
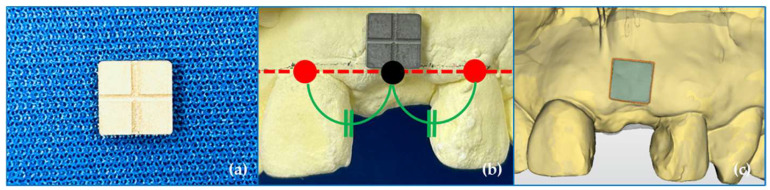
Establishing a region of interest. (**a**) A square stainless-steel guide (5 × 5 × 1 mm^3^). (**b**) A 5 mm square centered on the midpoint of the line connecting the cementoenamel junction of the two adjacent teeth (red line) was set perpendicular to the red line and toward the root apex. (**c**) A region of interest was projected onto the surface of the standard tessellation language (STL) data obtained before surgery (T0).

To establish a region of interest, a 5 mm square centered on the midpoint of the line connecting the cementoenamel junction of the two adjacent teeth (red line) was set perpendicular to the red line and toward the root apex. Before surgery, an impression was acquired according to the usual method, and a preoperative plaster model was created. A square stainless-steel guide (5 × 5 × 1 mm^3^) was used to set the region of interest (Figure 4). On the preoperative plaster model, a line was drawn connecting the lowest points of the cervical area of the two teeth adjacent to the missing tooth site. The stainless-steel guide was subsequently placed such that one side of the square was in contact, and digital scanning was performed. Using the matching software, the data obtained from the preoperative plaster model were superimposed with the STL data obtained before surgery (T0), and the square area of the stainless-steel guide was projected onto the data obtained before surgery (T0), and it was then used as the region of interest.

#### 2.4.2. Measurement of the Volumetric Changes

**Figure 5:**
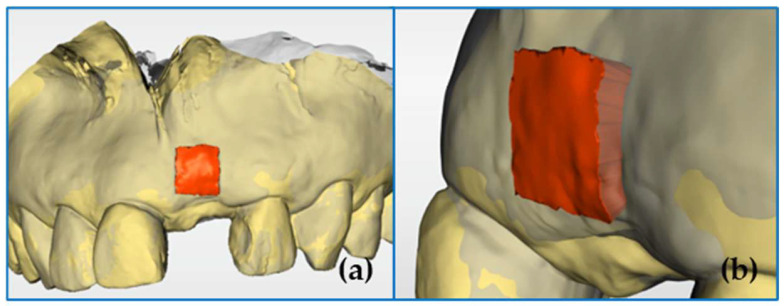
Measurement of the volumetric changes. Volumetric change in the region of interest, determined by superimposing the preoperative (yellow) and postoperative (gray) data. (**a**) Overall view, (**b**) Lateral view.

The STL data obtained after surgery (T1, T2, or T3) were superimposed with the data obtained before surgery (T0), and the volumetric change in the region of interest was measured using the analysis software. Within the region of interest, a line was drawn from each polygon vertex on the surface of the baseline (T0) STL data to the nearest neighbouring polygon vertex on the surface of the postoperative STL data, and the volumetric change was depicted as the 3D field formed by the lines (Figure 5).

#### 2.4.3. Measurement of the Cross-Sectional Dimensional Changes

**Figure 6:**
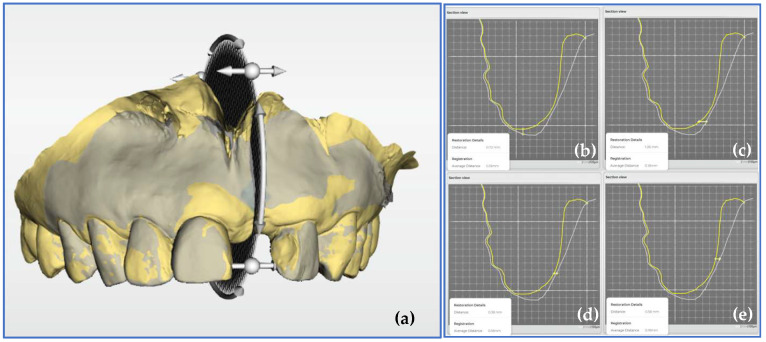
Measurement of the cross-sectional dimensional changes. (**a**) Setting of a cross-section orthogonal to the dental arch. (**b**) Horizontal dimensional change in the cross-section at 1 mm from the alveolar ridge crest. (**c**) Horizontal cross-sectional dimensional change at 3 mm from the alveolar ridge crest. (**d**) Horizontal cross-sectional dimensional change at 5 mm from the alveolar ridge crest. (**e**) Vertical cross-sectional dimensional change.

After superimposing the preoperative (baseline) and postoperative STL data, a cross-section orthogonal to the dental arch was created at the center of the region of interest. From the alveolar ridge crest on the preoperative data (T0), a line was drawn in the Y-axis direction, and the distance to the intersection with the alveolar ridge surface on the postoperative data (T1, T2, or T3) was defined as the vertical dimensional change (Figure 6). Furthermore, at 1, 3, and 5 mm from the alveolar ridge crest on the preoperative data (T0), a line was drawn in the X-axis direction from the alveolar ridge surface on the preoperative data (T0), and the distance to the intersection with the alveolar ridge surface on the postoperative data (T1, T2, or T3) was defined as the horizontal dimensional change.

### 2.5. Statistical Analysis

The volumetric and dimensional changes after guided bone regeneration (GBR) were statistically analyzed by analysis of variance (ANOVA) and post hoc comparisons with the Bonferroni correction (RStudio, version 2025.05.1; The R Foundation for Statistical Computing, Vienna, Austria). The significance level was set at *p* < 0.05.

## 3. Results

The participants included 13 patients, comprising 5 males and 8 females, with a mean age of 45.77 years (males: 42.80 years, females: 47.63 years). The sites of the missing teeth included the central incisor, lateral incisor, and the canine in nine, three, and one patient(s), respectively (Table 1). The causes of tooth extraction included root fracture, apical periodontitis, and unknown in eight, three, and two patients, respectively.

To assess intra-operator reliability, repeated measurements were performed twice by the same operator. The intraclass correlation coefficients [ICC(3,1)] for the three comparisons (T0–T1, T0–T2, T0–T3) ranged from 0.9968 to 0.9992, with narrow 95% confidence intervals (0.9895–0.9998), indicating excellent reproducibility of the measurements.

Prior to the comparative analyses, we confirmed the normal distribution of the residuals by the Shapiro–Wilk test (*p* > 0.05).

In the primary analysis, repeated-measures ANOVA with Greenhouse–Geisser correction revealed significant differences among the time points (*p* < 0.01). As a sensitivity analysis, we additionally fitted a linear mixed-effects model with a random intercept for subjects (Value ~ Condition + (1|Subject)). The mixed-effects analysis yielded consistent results with the ANOVA, confirming significant effects of time points (*p* < 0.01). The main significant contrasts (T1 vs. T2, T1 vs. T3) were identical in both methods, whereas slight discrepancies in *p*-values for borderline comparisons (e.g., T2 vs. T3) reflected differences in degree-of-freedom estimation (Greenhouse–Geisser vs. Satterthwaite approximation). These differences did not affect the overall interpretation.

### 3.1. Volumetric Changes (Table 2)

**Figure 7:**
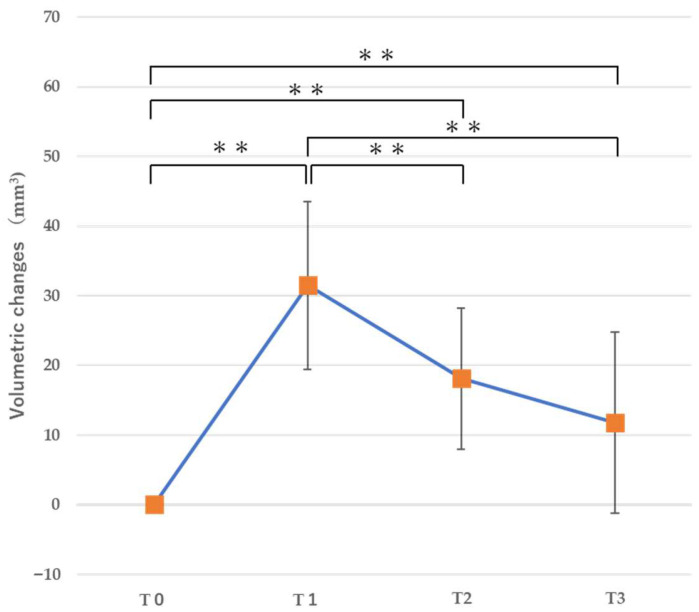
Volumetric changes in the region of interest. ** *p* < 0.01.

At all time points (T1, T2, and T3), a significant volumetric increase was observed compared to the baseline (T0). In particular, a significant increase at T1 (immediately after bone augmentation, mean: 33.53 ± 13.49 mm^3^) was observed, followed by a significant decrease at T2 (*p* = 0.0002 < 0.01). In addition, no volumetric change was observed from T2 to T3 (Figure 7), indicating stabilization of the augmented bone 6 weeks after bone augmentation.

### 3.2. Dimensional Changes in Cross-Section

#### 3.2.1. Horizontal Dimension (Table 3, Figure 8, Figure 9 and Figure 10)

**Figure 8:**
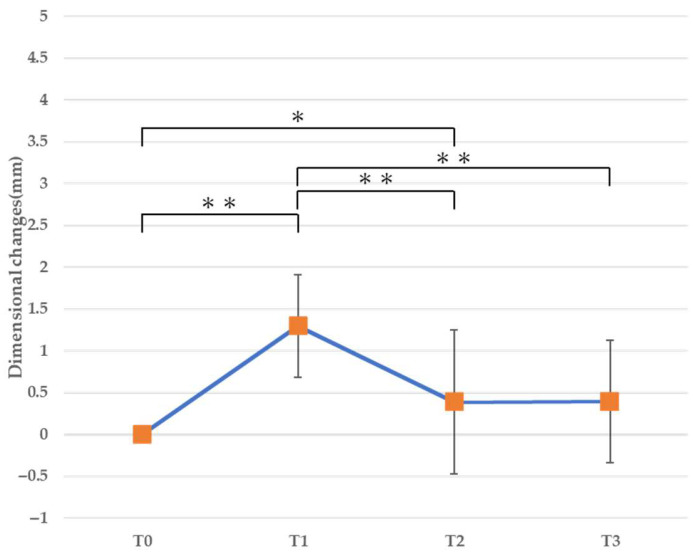
Horizontal cross-sectional dimensional changes (1 mm). * *p* < 0.05, ** *p* < 0.01.

**Figure 9:**
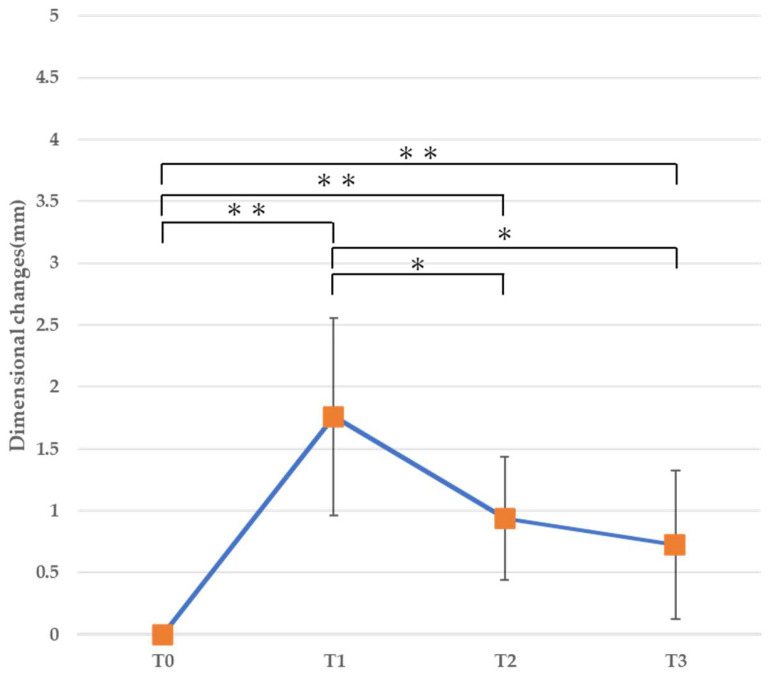
Horizontal cross-sectional dimensional changes (3 mm). * *p* < 0.05, ** *p* < 0.01.

**Figure 10:**
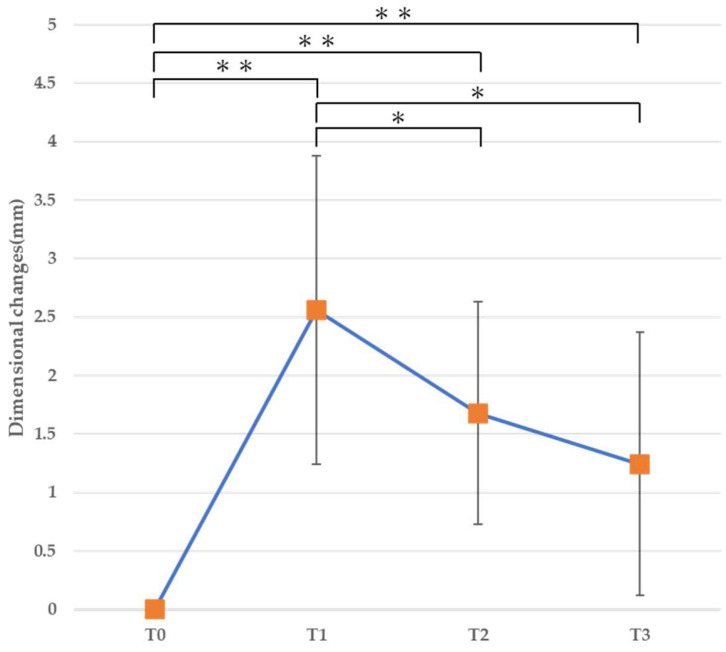
Horizontal cross-sectional dimensional changes (5 mm). * *p* < 0.05, ** *p* < 0.01.

At T1 (immediately after bone augmentation), a significant increase in the horizontal dimension was observed at all levels. At 1 mm from the alveolar ridge crest, a significant decrease was observed from T1 to T2 and from T1 to T3 (*p* < 0.01), and the horizontal dimension at T3 demonstrated no difference compared to before surgery (Figure 8). At 3 mm from the alveolar ridge crest, a significant increase was observed at all time points (T1, T2, and T3) compared to before surgery (*p* < 0.01), while a significant decrease was observed from T1 to T2 and from T1 to T3 (*p* < 0.05) (Figure 9). Similarly, at 5 mm from the alveolar ridge crest, a significant increase was observed at T1, T2, and T3 (*p* < 0.01), while a significant decrease was observed from T1 to T2 and from T1 to T3 (*p* < 0.05) (Figure 10).

**Table 3:** Dimensional changes in the cross-section.

	Horizontal dimensional changes in cross-section, mean (mm) ± SD			
		**T1**	**T2**	**T3**
H1 mm	Means ± SD	1.30 ± 0.61	0.39 ± 0.86	0.39 ± 0.73
	*p* value	0.0003	0.0002	0.5579
	95% CI	[0.85 1.67]	[−0.19 1.67]	[−0.15 0.83]
H3 mm	Means ± SD	1.76 ± 0.80	0.94 ± 0.50	0.72 ± 0.60
	*p* value	0.0006	0.0001	0.0305
	95% CI	[1.18 2.26]	[0.60 1.29]	[0.33 1.15]
H5 mm	Means ± SD	2.56 ± 1.32	1.68 ± 0.95	1.25 ± 1.13
	*p* value	0.017	0.0022	0.017
	95% CI	[1.60 3.40]	[1.09 2.38]	[0.53 2.07]
	Vertical dimensional changes in cross-section, mean (mm) ± SD			
		**T1**	**T2**	**T3**
Vertical dimensional changes	Means ± SD	0.65 ± 0.58	−0.21 ± 0.56	−0.18 ± 0.57
	*p* value	0.0056	0.0106	0.3063
	95% CI	[0.22 0.98]	[−0.59 0.18]	[−0.52 0.25]

Note: Preoperative data (T0) were set as the baseline.

#### 3.2.2. Vertical Dimension (Table 3, Figure 11)

The vertical dimension in the alveolar ridge crest demonstrated a statistically significant decrease (loss) from T0 to T1, from T1 to T2 (*p* = 0.0056 < 0.01), and from T1 to T3 (*p* = 0.0106 < 0.05). Dimension loss was observed in 9 of 13 patients (69.2%) who underwent horizontal augmentation.

Therefore, the cross-sectional dimensional change evaluation revealed that the height of the alveolar ridge decreased early after horizontal bone augmentation and that its width increased toward the root apex.

**Figure 11:**
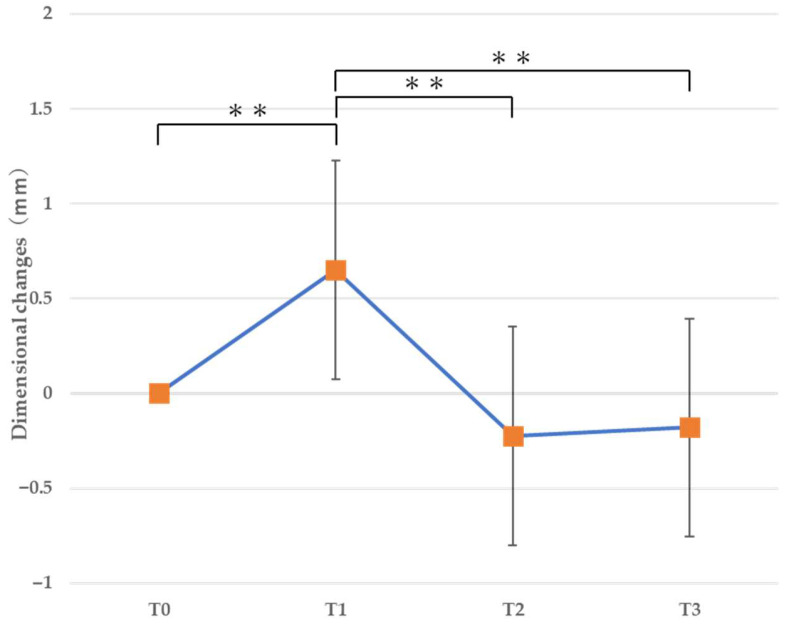
Vertical cross-sectional dimensional changes. ** *p* < 0.01.

## 4. Discussion

In this study, the largest volumetric gain was observed at 2 weeks after horizontal bone augmentation, followed by a marked decrease. Furthermore, the evaluation of cross-sectional dimensional changes showed that the alveolar ridge height decreased early after surgery and that its width increased toward the root apex.

Zhang et al. examined patients who underwent implant placement with horizontal GBR for one or two maxillary anterior teeth, and they used CBCT to investigate the effect of fixation of the resorbable membrane, as well as its methods, on the postoperative stability of hard tissues [41]. Immediately after and 6 months after surgery, they measured horizontal bone loss as the distance at the levels of 0–5 mm from the implant shoulder area toward the root apex, and they found that patients without membrane fixation exhibited a significantly greater horizontal bone loss than those with membrane fixation (mean: 1.04–1.25 mm). In the present study, the mean horizontal dimensional change in the alveolar ridge from 2 weeks to 3 months after surgery was 0.39–1.25 mm at the levels of 1–5 mm from the alveolar ridge crest, showing a change comparable to that in the previous study; however, differences were observed in the hard tissue, alveolar ridge, and measurement period. In addition, Arnal et al. examined the conventional method and the sausage technique in a staged GBR approach for the jawbone with knife-edge morphology (incisor area: three sites, canine area: three sites, premolar area: eight sites, molar area: two sites), and they used CBCT to evaluate the amount of horizontal bone augmentation at 2 mm from the alveolar ridge crest, as well as the amount of bone loss at 6 months after surgery [42]. The amount of horizontal bone augmentation was significantly less in the conventional method group (2.7 ± 1.8 mm; 83.2%) than in the sausage technique group (5.3 ± 2.3 mm; 216.8%). Additionally, the amount of bone loss was significantly reduced in the conventional method group (0.9 mm; 27.9%) compared with the sausage technique group (2.1 mm; 29.4%); however, no significant difference was observed in the rate of bone loss. Furthermore, Mertens et al. investigated the effect of wound closure in GBR on the stability of the graft materials in vitro using porcine mandibular bone, and they used CBCT to comparatively evaluate four different surgical procedures of bone grafting: Group 1, procedure using unfixed resorbable collagen membrane and granular xenograft material; Group 2, procedure using resorbable collagen membrane fixed with titanium pins and granular xenograft material; Group 3, procedure using non-resorbable titanium-reinforced polytetrafluoroethylene membrane and granular xenograft material; and Group 4, procedure in which screw-fixed autologous bone block and granular xenograft material were covered with unfixed resorbable collagen membrane [43]. Their results demonstrated a significant displacement of the graft material in Group 1 at 0–5 mm from the bone crest. The decrease rate in the horizontal direction after suturing was 63.5 ± 40.5% at 0 mm from the bone crest, 45.9 ± 30.9% at 1 mm from the bone crest, and 41.6 ± 25.3% at 2 mm from the bone crest, indicating a particularly large decrease in the horizontal direction near the alveolar ridge crest. The present study also observed a large temporal decrease in the alveolar ridge at a level close to the alveolar ridge crest, suggesting that the horizontal decrease may involve soft-tissue pressure attributed to wound closure with sutures.

Furthermore, according to a review by Munakata et al. [44], there have been no reports of differences in implant success rates or survival rates due to differences in graft materials used for vertical and horizontal bone augmentation. However, there is no doubt that these factors are important for achieving and maintaining osseointegration. In a systematic review of the effects of bone graft materials and membranes on horizontal and vertical bone augmentation by Troeltzsch et al. [45], the overall bone defect fill was 79.8% on average, with results ranging from 51.0% (artificial bone) to 85.8% (heterologous bone) with bone graft materials. In addition, with regard to horizontal bone augmentation, the average gain was 3.7 ± 1.2 mm, while the average gain with autograft mixed with allograft or xenograft was 4.5 ± 1.0 mm, which was significantly larger than those with artificial bone, such as HA and β-TCP (average 2.2 ± 1.2 mm). Furthermore, although there was no significant difference in the amount of new bone formation between autograft mixed with allograft and with xenograft (56.6%), the amount of new bone formation was large. In this study, autografts and xenografts were used, but it is necessary to investigate differences with other transplant materials in the future. Regarding membranes, non-absorbable titanium mesh showed significantly greater bone formation than collagen membranes, PTFE, and PLA/PGA, but titanium mesh had a higher incidence of early complications such as exposure and infection. In this study, we used absorbable porcine collagen membranes, but in recent years, relatively new absorbable membranes such as bovine pericardium membranes [46] and poly(l-lactic acid/ε-caprolactone) membrane [47] have been histologically examined and clinically applied.

In addition to their clinical handling properties, the biological behavior of graft materials and membranes is crucial for long-term GBR outcomes. Xenogeneic bone substitutes such as deproteinized bovine bone (e.g., Bio-Oss^®^) provide reliable osteoconductive scaffolding, whereas autologous platelet concentrates have been shown to release growth factors that modulate inflammatory mediators and promote angiogenesis and bone remodeling. These adjuncts may enhance wound stability and reduce early graft resorption. Furthermore, the choice of barrier membrane strongly influences the stability of augmented sites. Non-resorbable titanium-reinforced membranes and meshes are effective for maintaining space, but they are prone to complications such as exposure and infection. Conversely, collagen-based and pericardium-derived membranes offer favorable biocompatibility and resorption kinetics. Recent histological investigations, such as that by Bernardi et al. [46], demonstrated that bovine pericardium membranes not only provide mechanical support but also preserve structural integrity while allowing cellular integration, supporting their clinical use as a promising alternative to conventional membranes. Incorporating these considerations emphasizes that graft and membrane selection should be individualized to balance biological performance, complication risk, and clinical manageability.

Therefore, we will investigate differences in bone formation depending on the type of membrane and examine membranes suitable for alveolar ridge augmentation that combine high bone formation and shape maintenance.

However, very few studies have used intraoral digital scanning to investigate changes in the alveolar ridge. In a case series of ARP using a synthetic bone block, Park et al. performed digital scanning using IOS before surgery (baseline) and immediately after and 6 months after surgery, and they analyzed the volumetric changes in the alveolar ridge by superimposing IOS data (STL) obtained over time [48]. The measurements taken at a total of four sites (mandibular anterior tooth area in one patient and maxillary premolar area in three patients) demonstrated volumetric changes of 0.11 ± 1.08 mm^3^ from before surgery to immediately after surgery, 0.02 ± 0.8 mm^3^ from before surgery to 6 months after surgery, and −0.65 ± 0.82 mm^3^ from immediately after surgery to 6 months after surgery, demonstrating a postoperative volumetric decrease. In addition, Lee et al. examined patients undergoing implant placement immediately or soon after tooth extraction, and they used digital scanning to analyze the morphology of the surrounding soft tissues after the installation of the final implant superstructure and evaluated the volumetric changes separately for the upper and lower regions [49]. The upper region was defined as the soft-tissue region with no bone support, while the lower region was defined as the soft-tissue region supported by bone or bone augmentation. The results demonstrated a volumetric decrease at 12 months from the baseline (the time at which the final superstructure was installed) in both the upper region (patients with early implant placement: −5.40 ± 9.6 mm^3^; patients with immediate implant placement: −13.45 ± 11.83 mm^3^) and lower region (patients with early implant placement: −4.58 ± 8.72 mm^3^; patients with immediate implant placement: −10.50 ± 10.78 mm^3^), with no significant difference between the upper and lower regions. In the present study, we observed a large decrease in the vertical dimension of the alveolar ridge crest immediately after surgery, as well as in the horizontal dimension at 1 mm from the alveolar ridge crest, suggesting that the region was dependent only on changes in the soft tissues. Through the combined use of radiological evaluation, future studies need to clarify whether the volumetric change mainly involves soft or hard tissues.

Changes in the soft tissues can be evaluated in a non-contact manner using an IOS, and owing to the absence of pressure deflection attributed to an impression, digital scanning is considered a highly useful method for measuring the changes in the alveolar ridge, including soft tissues, compared to the conventional diagnostic methods using impression-based models [50]. In particular, since the marginal gingiva and the interdental papilla area are not supported by hard tissues such as bone, they are likely susceptible to deflection by impression pressure. Moreover, because this method enables the evaluation of the keratinized mucosa and the movable mucosa separately, it can measure changes in the peri-implant tissue and the keratinized mucosal width attributed to implant surgery or soft tissue augmentation, unlike model-based measurement methods [51]. Furthermore, because the method is non-invasive and time-efficient with no radiation exposure and places little burden on patients, it is considered more useful for measuring temporal changes than CBCT [52].

In this study, horizontal bone augmentation was performed in all patients, and we observed a large decrease in the vertical height of the alveolar ridge after surgery. The reason for the decrease in vertical alveolar ridge height is that Derakhshani et al. [53] performed 3D analysis using IOS to examine changes in soft tissue dimensions after implant placement and found that the conical connection showed significantly higher soft tissue proliferation and less resorption compared to the butt joint. Additionally, both studies reported that soft tissue resorption occurred around the implant and in the papillae, suggesting that soft tissue resorption is the causative factor. Moreover, considering the horizontal width of the alveolar ridge, we observed a large amount of alveolar ridge resorption at the alveolar ridge crest after surgery. These findings suggest that when performing horizontal bone augmentation, it is important to select a surgical procedure that maintains the vertical alveolar ridge and the horizontal volume at the alveolar ridge crest. Surgical procedures for maintaining the vertical alveolar ridge include connective tissue grafting during the non-loading period, the tenting screw technique using micro-screws or healing abutments, and the tent-pole technique [54,55,56]. Farias et al. examined 14 patients who underwent horizontal bone augmentation and implant placement surgery in the mandibular molar area using the tenting screw technique (27 sites); they used CBCT to radiologically analyze the bone width before and after surgery [57]. The mean bone width was 2.95 ± 0.75 mm before surgery and 7.15 ± 1.87 mm at 4 months after surgery, suggesting a significant increase (4.2 ± 1.26 mm); the authors reported that the tenting screw technique contributed to highly predictable bone augmentation. Lin et al. used CBCT to evaluate vertical increase in 44 patients who underwent vertical bone augmentation with titanium screws, freeze-dried bone allograft material, and xenograft material (50 sites) early after tooth extraction [54]. They found that the amount of vertical bone augmentation was greater at the site grafted with the combination of resorbable membrane and tent screws than at the site without tent screws. Additionally, the present study observed an increase in the horizontal width of the alveolar ridge as the distance from the alveolar ridge crest increased, suggesting the movement of bone graft material to the movable mucosal area after bone grafting. This is likely attributable to membrane and graft material displacement owing to wound closure or compression of the surrounding soft tissues and muscles. Our findings were similar to those of the aforementioned studies by Zhang et al. [41], Arnal et al. [42], and Mertens et al. [43], suggesting that fixation is necessary for bone maintenance at the alveolar bone crest. In addition, Felice et al. [58] conducted a clinical comparison of GBR using autogenous bone chips and porcine xenografts with titanium-reinforced PTFE membrane, titanium mesh, and titanium foil. They reported no difference in treatment outcomes or complication rates one year after implant placement, with an average height of 6.72 and mean average width of 7.69 mm. This suggests that the volume stability of grafting materials is critically important in GBR, and the use of membrane fixation or non-absorbable titanium membranes, as mentioned earlier, may also be beneficial to prevent graft loss or movement in the apical direction.

This study has certain limitations. The jawbone morphology at the missing tooth site was not classified before surgery, and differences based on the tooth type were not examined because of the limited sample size. Moreover, we did not examine the effect of the keratinized gingival width or soft tissue phenotype on changes in the alveolar ridge. Le et al. conducted a comparative study of the size of the vertical buccal bone defects and the treatment outcome of implant placement with simultaneous GBR, reporting that the amount of bone recovery was smaller with larger buccal bone defects (the amount of buccal thread exposure) [59]. Moreover, Galarraga et al. observed a negative correlation between the preoperative width of keratinized gingiva and the shrinkage rate of soft tissues in the surgical treatment for peri-implantitis [60]; these aspects should be investigated in detail in future studies. Furthermore, a radiological examination conducted by Chappuis et al. demonstrated a correlation between the thickness of soft and hard tissues [61]. These reports indicate the need for further detailed examinations. Furthermore, since this study was conducted using autogenous bone, xenografts, and porcine collagen membranes, no comparative studies were conducted with different ratios of xenografts [62] or with other graft materials, other membranes, or autologous platelet concentrate. In recent years, various bone graft materials and membranes have been developed and clinically applied, and we believe that detailed studies and considerations, including bone conductivity and bone induction, will be necessary in the future.

Further studies are warranted to investigate the stability of the alveolar ridge after GBR based on the number of missing teeth, the correlation between the volumetric change in the alveolar ridge before and after GBR and the width of keratinized gingiva, and whether the changes in the alveolar ridge after GBR are dependent on the hard or soft tissues, which can be examined by superimposing DICOM data obtained from CBCT images and STL data obtained from intraoral digital scanning. Furthermore, longer-term follow-up surveys are warranted, as the present study examined changes in the alveolar ridge only up to 12 weeks after surgery.

## 5. Conclusions

- The alveolar ridge width increased proportionally with the distance from the crest toward the root apex after horizontal bone augmentation.
- The vertical dimension of the alveolar ridge crest showed a marked decrease following augmentation.
- Intraoral digital scanning proved useful for the temporal and objective evaluation of alveolar ridge changes.
- To enhance volumetric stability at the crest and prevent graft displacement, membrane fixation with titanium pins or tenting screw techniques may be considered.
- Well-designed controlled clinical trials are required to validate these findings and establish definitive clinical recommendations.

## Figures and Tables

**Table 1:** Patient characteristics.

Patient Number	Age	Sex	Tooth	Cause of Tooth Extraction
1	48	Female	21	Root fracture
2	27	Male	21	Root fracture
3	63	Female	21	Unknown
4	43	Female	21	Apical periodontitis
5	47	Male	11	Apical periodontitis
6	46	Female	12	Unknown
7	52	Male	12	Root fracture
8	65	Female	22	Root fracture
9	40	Male	11	Root fracture
10	22	Female	11	Root fracture
11	48	Male	13	Apical periodontitis
12	42	Female	21	Root fracture
13	52	Female	11	Root fracture

**Table 2:** Volumetric changes in the region of interest.

	T1	T2	T3
Mean (mm^3^) ± SD	33.53 ± 13.49	18.08 ± 10.10	11.79 ± 12.99
*p* value	0.0006	0.0002	0.053
95% CI	[23.53 39.52]	[10.67 24.37]	[6.79 21.23]

Note: Preoperative data (T0) were set as the baseline.

## Data Availability

The raw data supporting the conclusions of this article will be made available by the corresponding author on request.

## Abbreviations

The following abbreviations are used in this manuscript:

STL	Standard Tessellation Language
CBCT	Cone-beam computed tomography
DICOM	Digital Imaging and Communications in Medicine
IOS	Intraoral scanner
3D	Three-dimensional
CAD/CAM software	Computer-Aided Design/Computer-Aided Manufacturing software
GBR	Guided bone regeneration
